# Case of Encephaloid Tumor of the Ovaries

**Published:** 1845-04

**Authors:** W. Butterfield

**Affiliations:** Little Fort, Ill.


*Case of Encephaloid Tumor of the Ovaries.* By W. BUTTERFIELD, M.D., Little Fort, Ill.

In the month of June, 1844, I was requested to visit Miss F., a resident of this county, "afflicted with a disease of the womb." The history of the case was, that between eight and nine months previous, shortly after recovering from a remittent febrile attack, she was seized with a sharp pain in the right iliac region. The proper antiphlogistic treatment was directed, and she soon was apparently well. Soon after this the patient observed a small, round, hardened tumor, at the point where she had experienced the pain. As she suffered but little inconvenience, no attention was paid to it, and her friends were not informed of its presence. For the first few months the tumor enlarged very slowly, and with but occasionally very slight pains. During the fourth month from the time it was first noticed, the tumor enlarged with more rapidity, it rose up out of the pelvic cavity and stretched over towards the mesial line. At this period she noticed a "swelling" in the left iliac region. This tumefaction, hard and round, increased in volume very rapidly; it extended upwards, and tended also towards the mesial line by degrees, until finally, in the language of the patient, "the swellings had met." From this time until a month or six weeks before I saw her, she had not experienced much pain; occasionally there would be so intense pains as to oblige her to resume the recumbent position for a few hours; at other times she followed her usual household pursuits. About four weeks before I saw her, she became subject to a very distressing dry cough; she also was frequently attacked with excessive paroxysms of dyspnœa; she began to experience general pains throughout the abdomen, and was confined to her room for the most of the time. During one of her paroxysms of dyspnœa, I was called to her.

Upon examination of the abdomen, I discovered in the umbilical region a large, hard, uneven tumor, which seemed to have prolongations on each side, which dipped down into both iliac regions. The main prominence could be traced inferiorly to within three inches of the symphyis pubis. Superiorly it was perceptible to a short distance above the umbilicus. One or two hard lobular projections, of the size of an orange, slightly elastic, could be felt on the centract tumor. Fluctuation was perceptible at several points. The diagnosis was an encysted tumor involving both ovaries. She had emaciated a good deal, the pulse was at 100 and quite weak, the appetite was very slight, the bowels were costive, but easily acted upon, and the urine was scanty and often bloody. Until within a period of six weeks from the time I first saw her she had menstruated regularly. Of the treatment little is to be said. The secretions as far as possible were regulated, internally and externally. Iodine and other alternatives were freely used. Two weeks before death, anasarca of the inferior extremities set in. Nine days before her decease she was tapped, and three quarts of a thin serous fluid withdrawn; between the operation and the period of her death, probably 7 or 8 quarts in addition oozed through the puncture. On the 4th of August following, death took place.

An examination was had four hours after death. An incision was made from the ensiform cartilage to the symphysis pubis, intersecting a transverse section. Withdrawing the integuments, the dissection was continued through the peritoneum, which was thickened and closely adherent to the diseased mass beneath; separating these adhesions there was exposed a large tumor involving both ovaries, and filling nearly the entire abdominal cavity. The broad ligaments were so closely connected with the disease as to render it impossible to trace them out. The round ligaments were densely united to the tumor, and from them large vessels entered the substance of the tumor. The fundus of the uterus was united to the tumor by a cellular membrane which was freely supplied with large vessels. The walls of the uterus were somewhat thinckeed, in other respects that organ was normal. The largest portion of the tumor was slightly to the right of the mesial line, so that we may suppose that the disease sprung from the right ovary, and after a certain period the left became involved. Above, the tumor lay in connection with the stomach, liver, and spleen. The bowels were forced upwards and backwards, and were agglutinated together by a cellular membrane which was spread over the interior surface of the tumor. This membrane was traversed in all directions by large vessels, some of them being of the size of a goose quill. Its surface was studded over with innumerable small, hard, round points, the texture of which corresponded to that of the tumor. The mesenteric glands were enlarged, slightly reddened and somewhat softer than natural. The colon was less than one half of the normal size. The liver which lay in connection with the tumor was enlarged, and its inferior half presented the same appearance externally, and on incisions being made into its texture, proved it to have taken on the same degeneration as the ovaries. The tumor was of a greyish white color, hard and slightly elastic. Its anterior surface was composed of a series of lobular projections, from the size of an orange down to that of an hickory nut. Upon removing the mass from its position, a section being made through its centre, exposed a cyst containing one quart of semi-gelatinous fluid of a pale yellow color. No communication could be found between this cavity and the peritoneal sac. Sections being made of the tumor proved it to be of the class styled by Lænnec and Prof. Carswell, *encephaloid*, cellular in its texture—the cells being produced by the passage of fibrous bands. Many of these cells were softened, containing fluid resembling cerebral matter. The external surface was more dense, resembling the class of scirrhus growth. After the evacuation of the fluid, the tumor was found to weigh five pounds and seven ounces.

