# Errata

**Published:** 1845-05

**Authors:** 


ERRATA.—In the Case of Encephaloid Tumor in our last, for "centract" read "central," for "alternatives" read "alteratives," and instead of "the secretions as far as possible, were regulated internally and externally," read "Internally and externally, iodine," &c.—ED.

